# Chronic lymphoplasmacytic villonodular proliferative synovitis in a 10-year-old Jack Russell Terrier dog

**DOI:** 10.1080/23144599.2020.1842038

**Published:** 2020-11-18

**Authors:** Tafara Mapuvire, Erick Kandiwa, Pricilla Mbiri, Alaster Samkange, Oscar Madzingira, Borden Mushonga

**Affiliations:** a Surgivet Veterinary Consultancy, Johannesburg, South Africa; b Biomedical Sciences Department, School of Veterinary Medicine, Faculty of Agriculture and Natural Resources, Neudamm Campus, University of Namibia, Windhoek, Namibia; c Production Animal Clinical Studies Department, School of Veterinary Medicine, Faculty of Agriculture and Natural Resources, Neudamm Campus, University of Namibia, Windhoek, Namibia; d Department of Animal Health, School of Veterinary Medicine, Faculty of Agriculture and Natural Resources, University of Namibia, Katima Mulilo, Namibia

**Keywords:** Dog, lymphoplasmacytosis, villonodular, proliferative synovitis, stifle, lameness

## Abstract

We describe a case of chronic lymphoplasmacytic villonodular synovitis (CLPVNS) associated with cranial cruciate ligament (CCL) disease in a 10-year-old spayed Jack Russell Terrier bitch. The bitch was presented to a veterinary clinic with severe, non-weight bearing, acute left hindlimb lameness. The bitch had previously been treated surgically for stifle CCL disease of the same joint, using the lateral fabellar suture (LFS) technique. Since the treatment, the patient had a history of intermittent left hindlimb non-weight bearing lameness that was manageable with nonsteroidal anti-inflammatory drugs (NSAIDs). Palpation and manipulation of the affected stifle elicited severe pain. There were no other clinical or orthopaedic abnormalities. Orthogonal radiographs of the affected stifle revealed moderate degenerative joint disease and osteolytic lesions on the lateral aspect of the lateral femoral condyle and the head of the fibula. A fluid aspirate from this joint was negative for bacterial growth on culture. Cytology results were suspicious for CLPVNS. Exploratory arthrotomy, synovectomy, debridement and lavage of the affected joint were performed. Bone and synovial membrane biopsy samples of the joint were obtained and submitted to a laboratory for a histopathological confirmatory diagnosis. CLPVNS was tentatively diagnosed by cytology, and confirmed by histopathology of biopsy samples. This case report highlights the importance of checking for CLPVNS in dogs with lameness associated with CCL disease, as reports show it to be underreported or misdiagnosed.

## 1. Introduction

Villonodular synovitis is a diffuse inflammatory and/or proliferative condition of any joint synovial membrane, with an immunological aspect [1–4]. The condition is characterized by a reddish-brown colouration, hypertrophy and villous or non-villous proliferation of the synovial membrane [5–7]. When chronic and associated with plasma cell and/or lymphocyte infiltrates, the condition is known as chronic lymphoplasmacytic villonodular proliferative synovitis (CLPVNS) [8,9]. CLPVNS has been described in humans [6,9–11], dogs [1,3,12–15], horses [8] and giraffe [16].

In dogs, CLPVNS can affect any joint of any limb [11,17,18] but has more often been reported in the stifle joint, where an association has been established with cranial cruciate ligament (CCL) disease [1,3,12–15]. Though reportedly uncommon [13], CLPVNS has been reported to occur in 51% of dogs with cranial cruciate ligament disease [1] which, in turn, occurs in about 0.56–1.19% of dogs in the United Kingdom and US [19,20]. It is, however, frequently misdiagnosed [9].

The aetiology of villonodular synovitis (VS) is uncertain [6,12,14]. Degenerative [1,2,19,21,22], immunopathological [3,23], reactive inflammatory [6,15], neoplastic mechanisms [11,24] or both neoplastic and reactive inflammatory mechanisms [16] have been suggested as possible causes.

Villonodular synovitis is usually confirmed by histopathology [3,12–15,25–27] and cytology [1,28], especially if arthroscopic, radiological [5,6], physical examination [19], and arthrotomy findings are suggestive of the disease. More sophisticated techniques such as magnetic resonance imaging [23], computed tomography [11], biomarker assays and PET-SCAN [19] are also useful techniques for the diagnosis of VS.

Clinical findings are usually consistent with degenerative joint disease and include severe pain and lameness of the affected limb. Severe pain is elicited on palpation and manipulation of the affected joint [13]. The condition is normally treated by arthrotomy followed by synovectomy and post operatively managed with steroids or non-steroidal anti-inflammatory drugs (NSAIDs) and radiation therapy [26].

Development of CLPVNS in the stifle joint associated with CCL disease has been reported from western Europe [11,15,19,25,29], Scandinavia [12,13] with a vast majority of reports from the United States [1,3,14,23,27,30,31].

After management of a CLPVNS case in Johannesburg, South Africa, search for literature on the disease yielded only a limited number of reports. The authors discovered that CLPVNS associated with CCL rupture was a common and yet frequently misdiagnosed and underreported condition [9]. The current case report highlights the need to check for CLPVNS in cases of CCL disease in dogs. The report briefly describes the clinical presentation, management and outcome of CLPVNS secondary to CCL rupture.

## 2. Case description

A 10-year-old spayed Jack Russell Terrier bitch weighing 8.6 kg was presented with a progressive, acute onset, severe, left hindlimb lameness one day before clinical presentation. The dog had a history of CCL disease of the same stifle joint, 19 months prior to presentation. The CCL disease had been surgically managed using the lateral fabellar suture technique. Medically, the bitch was managed preoperatively with meloxicam (Metacam®, Boehringer Ingelheim) at a loading dose of 0.2 mg/kg subcutaneously and maintained postoperatively on carprofen (Rimadyl®, Zoetis) at 2.18 mg/kg orally twice a day for 5 days. In the months following lateral fabellar suture stabilization, there was occasional, mild and intermittent lameness of the left stifle that responded well to rest and short courses of oral carprofen (Rimadyl®, Zoetis) at 2.18 mg/kg twice a day.

On presentation of the bitch for acute lameness, physical examination revealed that all the parameters were normal, but the left stifle joint was very painful and swollen. Further orthopaedic findings were otherwise unremarkable. The differential diagnoses for the condition included, but were not limited to, peri-prosthetic infection/inflammation, trauma, degenerative joint disease, meniscal pathology, osteosarcoma and synovial sarcoma.

**Figure 1.**
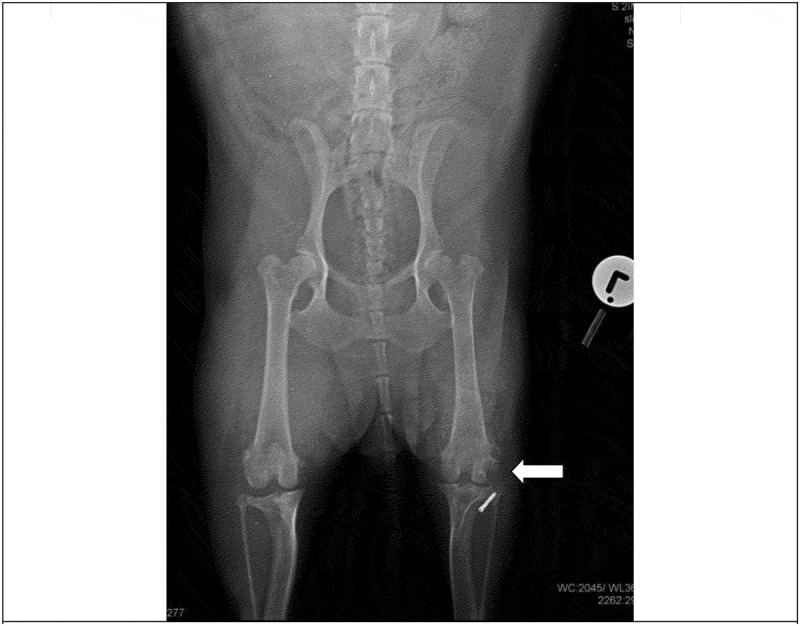
Ventro-dorsal radiographic view of the pelvis and cranio-caudal radiographic view of both stifles at the time of presentation. Note the osteolytic lesions on the lateral femoral condyle and head of the fibula of the left pelvic limb, reduced joint space and soft tissue swelling around the stifle (white arrow). The metallic crimp clamp used to secure the lateral fabellar suture nylon prothesis is visible on the lateral aspect of the lateral tibial condyle

**Figure 2.**
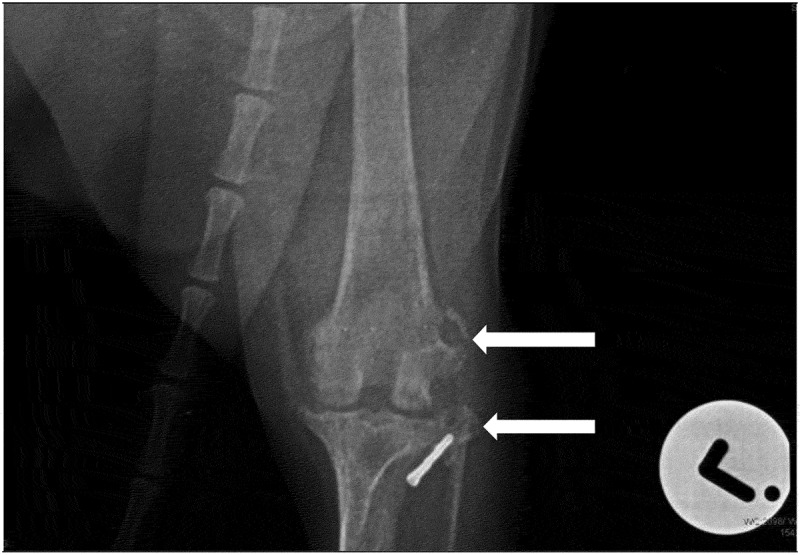
Cranio-caudal radiographic view of the left stifle at the time of presentation. Note the metallic crimp clamp used to secure the lateral fabellar suture nylon prosthesis on the lateral aspect of the lateral tibial condyle. Osteolytic lesions on the lateral femoral condyle and the head of the fibula are evident (white arrows)

**Figure 3.**
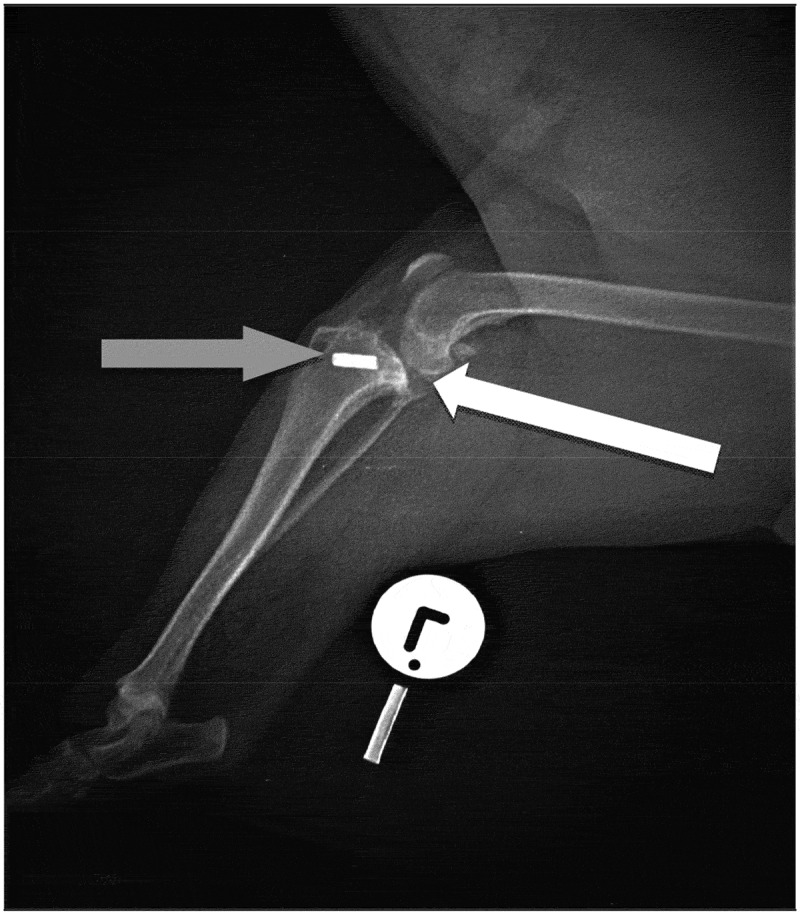
Lateral radiographic view of the left stifle. Note the metallic crimp clamp used to secure the lateral fabellar suture on the proximal tibia (grey arrow). Moderate degenerative changes are also evident in the joint (white arrow)

A set of orthogonal radiographs of the pelvis, hip and stifle joints (Figures 1–3) were taken under light general anaesthesia induced with intravenous medetomidine (Domitor®, Zoetis) at 12 µg/kg and intravenous propofol (Propoven®, Fresenius Kabi AB) at 3.5 mg/kg. Evaluation of the orthogonal radiographs of the left stifle revealed osteolytic lesions and periosteal reaction on the lateral aspect of the lateral femoral condyle and the proximal aspect of the head of the fibula (Figures 1 and 2). In addition, there was radiographic evidence of moderate degenerative joint disease of the same joint (Figure 3).

Under the same light general anaesthesia, about 0.3 millilitres of sero-sanguineous, reddish orange, slightly turbid joint fluid with reduced viscosity was also aspirated from the left stifle joint. The sample was submitted to a laboratory for cytology and culture. Cytology indicated that the joint fluid from the left stifle had a blood admixture with moderately high cellularity of nucleated cells, comprising predominantly of macrophages and nucleated neutrophils, in a proteinaceous precipitate displaying windrowing of cells. No microorganisms were observed. Further, the report noted that the joint fluid was characterized by mild anisocytosis, cytoplasmic basophilia with eccentrically placed nuclei, mild to moderate anisokaryosis, fairly mature chromatin, inconspicuous nucleoli, some mitotic figures and some multinucleate cells. Microbial culture of the fluid showed no growth after an initial four days followed by a further 48 hours of incubation under aerobic and microaerophilic atmospheres. A tentative diagnosis of CLPVNS was arrived at on the basis of history, signalment, physical examination, radiographic evaluation, clinical findings and cytology. Postoperatively, the bitch was managed with meloxicam (Metacam®, Boehringer Ingelheim) at a loading dose of 0.2 mg/kg subcutaneously and maintained with oral robenacoxib (Onsior®, Elanco) at 1.2 mg/kg once daily and oral gabapentin (Epleptin®, Ranbaxy) at 12 mg/kg twice daily for 5 days.

Based on the joint fluid cytology, exploratory arthrotomy [11], with the objective of synovectomy [9], joint debridement; and biopsy sample collection for the purposes of a confirmatory diagnosis was performed under general anaesthesia 4 days after receipt of joint fluid cytology and culture results. General anaesthesia was induced intravenously using diazepam (Pax®, Intramed) at 0.35 mg/kg, buprenorphine (Temgesic, Reckitt Benckiser) at 17.5 µg/kg and propofol (Propoven®, Fresinius Kabi) at 5 mg/kg. The dog was intubated to facilitate maintenance of general anaesthesia with intratracheal inhalation of a mixture of oxygen and 2% isofluorane (Isofor®, Safeline Pharmaceuticals) [32].

The left stifle was accessed using a routine craniolateral parapatellar arthrotomy approach as described by Pierrmattei and Johnson [31]. Grossly, the synovial membrane was reddish-brown in colour showing a diffusely thickened and villous appearance accompanied by mild quantities of proteinaceous debris. Bone and synovial membrane biopsy samples of the affected area were collected just before the excision of excess synovial membrane. In addition, the cruciate ligament repair nylon prosthesis was explanted during the exploratory arthrotomy procedure. After generous lavage of the joint with sterile saline, the surgical site was closed routinely using polydioxanone 4/0 suture (MacZyn®, Scimitar). No cutaneous sutures were placed. The procedure lasted approximately one hour. The bitch was discharged on the day of surgery and continued on robenacoxib at 1.2 mg/kg orally once daily and gabapentin at 12 mg/kg orally once daily for 5 more days.

The histopathological report of the biopsy samples from the laboratory indicated a diffuse thickening (hypertrophy) of the synovial membrane, forming non-proliferative papillary-type exophytic fronds covered by plump reactive synoviocytes. There was an increased vascularity of the sub-intimal layer. The sub-intimal layer was also infiltrated with single or multiple neutrophilic, lymphocytic and plasmacytic cell populations.

## 3. Discussion

The signalment, clinical signs, history, radiographic and cytological findings of the current case are suggestive of CLPVNS as described in literature. The diagnosis of CLPVNS was confirmed by histopathology. The case reported herein was that of an older spayed bitch which fits with the profile of CLPVNS cases reported in literature [1,8,13,14]. It is notable, however, that CLPVNS has not been previously described in a Jack Russell Terrier. In fact, the condition has, often times, been reported in large breed dogs [1,14] and occasionally in both large and small breed dogs of different sexes [20,29]. The history of CCL disease and clinical signs of unilateral, painful and swollen stifle, and non-weight bearing intermittent lameness [9,11] observed in the current case further reinforced the suspicion of CLPVNS.

Radiographic signs of degenerative joint disease typical of CLPVNS in this case have also been described in previous cases [1,5]. The increased joint effusion, narrowing of joint space and osteophyte formation observed in the current case have also been described in previous cases of CLPVNS [1,5,8,9,17,33]. Radiographic evaluation of the left stifle joint in the current case showed all the signs described above except for subchondral bone lucency. The results of radiographic evaluation also showed lysis of the lateral femoral condyle and the head of the fibula, signs probably related to the prosthesis that was used to stabilize the joint instability caused by cranial cruciate ligament rupture. However, CLPVNS has been reported secondary to cruciate ligament disease even in the absence of previous surgical intervention [30].

Furthermore, the sero-sanguineous appearance of joint fluid and the reddish-brown discolouration of the synovial membrane reported in this study has also been described in cases of villonodular synovitis by other studies [6,8,16]. The negative culture, high cellularity, mitotic figures, appearance of red blood cells, mild nucleated neutrophilia and abnormal mononuclear cells on microscopy and the proteinaceous precipitation of the joint fluid [1,28,34], though not pathognomonic for CLPVNS, is suggestive of the condition. Not much should be read into the negative synovial fluid culture results, as this method has a low sensitivity. However, histology of the synovial fluid smears was also negative, thus pointing to a non-infectious synovitis [28].

In the current case, the histopathological picture of a diffusely hypertrophied synovial membrane filled with plump reactive mononuclear cells (synoviocytes and macrophages) increased vascularity and infiltration of the sub-intimal layer with neutrophils, lymphocytes and plasma cells of the synovial membrane gave a definitive diagnosis CLPVNS especially when considered together with clinical examination findings, history, signalment, radiography, cytology and gross findings. This typical histopathological picture is consistent with descriptions of CLPVNS by previous studies [1,3,12,14,15,25–27].

Synovitis was, however, either missed or was not assessed at the time of the surgical procedure to correct CCL in the current case. According to Hulse et al. [35], synovitis may be missed when CCL correction is performed as it is not routinely assessed in the procedure. In several studies, CCL rupture and CLPVNS have been reported to occur in association with each other [1,3,12,14]. The actual sequence of events and the cause-effect relationship between the two conditions, however, remains elusive [2,7,18,36]. The rupture of the CCL on the left stifle was either a sequel to an acute traumatic event or repetitive micro-injuries associated with stifle joint instability [2,8,9,13]. It has been demonstrated that cell mediated and/or humoral immune mechanisms against type I and type II collagen fibres in the CCL and joint cartilage are involved in synovitis associated with rupture of the CCL [1,12,34].

The immune response within the synovial joint involves the systemic elaboration of acute phase reactants cytokines and immunoglobulins into the circulatory system and the affected joint [1,15,22]. These pro-inflammatory substances recruit inflammatory cells such as macrophages, neutrophils and lymphocytes that amplify inflammation in both the ipsilateral and contralateral stifle joints and damage the cruciate ligaments of both joints [3,23]. Joint haemorrhage resulting from chronic injuries due to joint instability [8] and/or chronic inflammation or autoimmune response [15,22,23,26] which initiated the CLPVNS can explain the haemosiderin-laden macrophages and the reddish-brown discolouration of the synovial membrane. The pathology may even extend to the caudal cruciate ligaments of both stifle joints [21].

Contrary to reports that only well circumscribed lesions have a good prognosis in man and horses [8], the case reported herein recovered uneventfully and was clinically normal at the time this report was compiled (six months after surgery). The dog also responded well to explantation of the cruciate nylon leader line prosthesis and a course of NSAIDs, consistent with what is described in literature.

In this current case report, it is impossible to ascertain whether CLPVNS developed secondary to surgical management CCL or was actually the cause of CCL rupture that might have been missed at the initial stage of CCL diagnosis and treatment. However, the association of the two conditions is confirmed. CLPVNS was confirmed by histopathology, surgically treated by synovectomy, debridement and lavage. The intermittent post-surgical lameness was successfully managed with carprofen and oral gabapentin. The treatment plan brought about complete recovery of the bitch within six months of treatment. Finally, practitioners are advised to look out for CLPVNS whenever CCL disease is suspected or diagnosed.
